# M. Richerand's Progress of Surgery—Illiberality of the French Critics

**Published:** 1826-07

**Authors:** 


### FRENCH CRITICISM; OR, NATIONAL FEELING.*

In the year 1750, Mr. Samuel Sharp published his "Critical Enquiry into the present State of Surgery," a work which obtained for the author great commendation and extension of fame, while it was of considerable benefit to the public at large. It is professedly on the model of our countryman, and for the same avowed purpose, that M. Richerand has come forward with his present work. But the fate of this performance promises to be very different from that of Mr. Sharp's Enquiry. M. Richerand seems to have given grievous offence to his countrymen, and to have so wounded their national pride that, had he lived in the good old times of the Revolution, he would have probably gone to the CONCIERGERIE, if not to the GUILLOTINE. Our French brethren seem to be peculiarly irritable when any credit is given to foreigners, which one would suppose they considered as a detraction from their own merits. They cannot relish the idea that improvement in either medicine or surgery should originate or become perfected beyond the limits of their own dear country. Doubtless there is no land without its prejudices and partialities, England cannot boast of immunity from those weaknesses; but we think any English surgeon might write a book and extol foreigners to the neglect of his own com-patriots, without drawing such a hornet's nest about his ears as M. Richerand has done. He might laud the Otaheitean mode of venesection by means of an arrow, to that practised in England:—He might even prefer the Barbary operation of amputation by a sharp axe, the bleeding stump being plunged into a Bamboo case filled with bituminous substances to save tenacula and future dressings, to the most improved methods by Liston and Syme, without drawing down the vengeance of a host of critics on his head. But this might not be in France. It would be a crime against national pride, and would be punished by a hundred brochures—nay, the author would certainly be *denationalised!*

Under the mask of paying respect to our countryman, the French critics have indirectly cast severe reflections on M. Richerand. Sharp, say they, was *correct and faithful* in his exposé—moderation and justice presided over his criticisms—and rigid impartiality was the soul of his judgments. Sharp sought not to tarnish the reputation of his masters—envy did not lead him to defame his cotemporaries—nor ravish from his com-patriots the honour of discoveries, in order to heap them on foreigners. Sharp wrote without anger or hatred—he had nothing in view but the interests of science—he put individuals out of sight when he looked at error, or sought for truth. Finally, he possessed the rare talent of compressing a great deal of matter in few words. Of course it is meant that M. Richerand's portrait is the reverse of this.

The first tangible charge against our unfortunate author is blindness to the merits of Bichat. The latter simplified and perfected the instru-

---

* Histoire des Progrès recens de la Chirurgie; par M. Le Chevalier Richerand, M. D. &c. &c. 8vo. 1826.

ments employed in trephining the cranium. Richerand has passed over these improvements in silence. Next it is said that Desault's method of operating for fistula lachrymalis was practised in France till the time of Dupuytren, and was even recommended by Richerand himself in the earlier editions of his Chirurgical Nosography. Dupuytren, however, conceived the idea of re-establishing the nasal canal by the introduction of a golden canula into the tract of the duct, by which an effectual cure was quickly performed. Reflecting on the general want of success attending this method as originally invented by Foubert, Dupuytren found that it was owing to the smallness and the shape of the canula hitherto employed. He enlarged the size, and so modified the shape that the instrument could be retained a sufficient length of time to effect a cure in as many days as it formerly required months. M. Richerand now praises the operation by the canula, but says nothing of Dupuytren who brought it to perfection.

Riolan, say the critics, advised, two centuries ago, the perforation of the tympanum, in cases of congenital deafness—and Cheselden observed, that this operation might be resorted to in certain diseases of that membrane. Still later, Julien Bousson recommended the operation where the interior of the ear was filled with pus. M. Richerand is accused of attributing the invention of this operation to our celebrated countryman Sir Astley Cooper. Now as it does not appear that the operation was ever *performed* before Sir Astley performed it, we really see no heinous crime in giving him the credit of the discovery. No man in this country will accuse Sir Astley of hunting through old musty volumes for *hints*, when the *book of nature* is so widely spread before him, and whilst his intellects remain to explore that fertile source of knowledge.

It appears that M. Itard has made an alteration in the *shape* of the instrument for perforating the membrana tympani, and the French critics are in a rage that M. Richerand should not have taken away all credit from Sir Astley, and wreathed the laurel round the brows of their countryman—M. Itard! *risum teneatis?*

The operation of staphyloraphia, as practised by Roux, has been claimed by Gräefe, of Berlin. M. Richerand is blamed, of course, for not deciding in favour of his countryman. It appears rather awkward indeed for M. Richerand that, last year, in the public section of surgery, he gave credit to M. Roux for the invention of the operation in question; but it is highly probable that he was ignorant of Gräefe's priority, for German literature is confessedly little known in the French medical world.

On the subject of lithotomy M. Richerand is accused of not doing justice to Dupuytren; and on that of aneurism, the honour of tying the artery above the tumour, without opening the sac, is rifled from John Hunter by the French critics, and given to M. Anel, who, 75 years previously, *proposed* this operation! M. Richerand is execrated, of course, for placing Hunter as the founder of this operation—" car Hunter n'a fait que modifier *legerement* le methode inventé par Anel." What we said of Sir Astley Cooper, respecting the operation of puncturing the membrana tympani, will doubly apply to John Hunter. Little did he know of Anel's proposal, we may be certain.

Spare ligatures have long been laid aside in this country, in the tying of arteries, though it is well known that, till very lately, if not till the present moment, not only were spare ligatures left around arteries in France, but the most clumsy interventions were used to keep the thread from cutting the coats of the vessel. Yet M. Richerand is anathematised for saying that the British surgeons are entitled to the praise of having simplified and rendered more safe the operation of tying arteries for aneurism!

That M. Richerand has written some passages under the influence of personal hostility to certain of his cotemporaries, especially M. Dupuytren, is not improbable; and that he has been guilty of inconsistency on several occasions, we are ready to admit; but that his liberality to the surgeons of other countries, particularly of England, should deserve such abuse and execration as are poured upon him from all quarters, we cannot believe. The French are acknowledged to be a vain nation, and the medical portion is not exempt from the general ruling passion. Their self-love is wounded, and they have carried their indignation to the highest pitch of extravagance, by which, in exposing the passion under which Richerand wrote, they have very conspicuously portrayed their own. Nothing can justify the hideous character which has been drawn both of Richerand and his book: "Un livre infidéle parsque des faits importans y sont omis ou presentés sous un faux jour; un livre partial, puisqu'il renferme d'injustes critiques et des eloges non merités; un livre dans lequel les interets sacrés de la verité sont incessamment sacrifiés à des inimitiés personelles; un livre enfin, dans lequel l'auteur déguise avec un art infini sous le semblant du vrai les faussetés les plus palpables." Such a passage against such a man as Richerand does not honour to the French press.

We have glanced over the work so severely criticised, and find it to contain a fair exposé of the actual state and recent progress of chirurgical knowledge—not vouching, however, for the strict impartiality of the author as to the origin of certain improvements. Partiality or impartiality, in this respect, only concerns individuals—to the public it is of comparatively little consequence who are the inventors of improvements, provided they be actually such. A regular review of M. Richerand's work would be unnecessary; but we shall have occasion to notice some parts of it while treating of chirurgical subjects in different parts of the Journal.

